# EHMTI-0259. Demonstrational project: to develop, implement and test an educational model for better headache-related primary health care

**DOI:** 10.1186/1129-2377-15-S1-D3

**Published:** 2014-09-18

**Authors:** M Braschinsky, S Haldre, M Kals, A Iofik, A Kivisild, J Korjas, S Koljal, Z Katsarava, T Steiner

**Affiliations:** 1 Neurology Clinic, Tartu University Hospital, Tartu, Estonia; 2 Estonian Genome Centre, Tartu University, Tartu, Estonia; 3 Faculty of Medicine, Tartu University, Tartu, Estonia; 4 Headache Centre Department of Neurology, University of Duisburg-Essen, Essen, Germany; 5 Department of Neuroscience, Norwegian University of Science and Technology, Trondheim, Norway

## Introduction

While headache disorders are under-recognized, under-diagnosed and undertreated everywhere in the world, a principal factor is lack of knowledge among health-care providers, especially those in primary care.

## Aim

To develop, implement and test an educational model for improving headache-related primary health care.

## Methods

The study was performed in the town of Põlva, Estonia, among 6 general practitioners (GPs) with a patient base of 12500. We made retrospective base-line and prospective outcome observations using the same measures, separated by the intervention. The primary outcome measure was the referral rate to specialist care, selected because it was objective and verifiable, and had economic impact. Secondary outcome measures included GP-made headache diagnoses, treatments recommended/initiated, investigations performed and several patient-related outcomes. The intervention was a 2-day educational course based on EHF recommendations.

## Results

Baseline review included 490 case records; prospective analysis included 295. Overall referral rate changed insignificantly (from 38% to 35%; p=0.19). However, other measures showed marked improvements. There was a strong shift from using non-specific headache diagnoses towards specific (39% to 13%; p<0.0001); diagnosis of precise migraine subtypes increased from 8% to 38% (p=0.0005). Headache treatment initiation rate increased from 58% to 81% (p<0001). (Unnecessary) investigation rate in patients with primary headache disorders fell from 26% to 4% (p<0.0001).

## Conclusions

This is the first direct demonstration that well-structured education of GPs in headache improves outcomes, with fewer unnecessary investigations, and is probably cost-saving. Clearly the study needs replicating, and there is work to do to develop the methodology of these studies.

No conflict of interest.

